# Association Analysis for Some Biochemical Traits in Wild Relatives of Wheat under Drought Stress Conditions

**DOI:** 10.3390/genes13081491

**Published:** 2022-08-21

**Authors:** Alireza Pour-Aboughadareh, Omid Jadidi, Lia Shooshtari, Peter Poczai, Ali Ashraf Mehrabi

**Affiliations:** 1 Seed and Plant Improvement Institute, Agricultural Research, Education and Extension Organization (AREEO), Karaj P.O. Box 31587-77871, Iran; 2 Department of Plant Breeding and Biotechnology, Science and Research Branch, Islamic Azad University, Tehran P.O. Box 14515/775, Iran; 3 Department of Plant Breeding and Biotechnology, Kermanshah Branch, Islamic Azad University, Kermanshah P.O. Box 67146, Iran; 4 Botany Unit, Finnish Museum of Natural History, University of Helsinki, P.O. Box 7, FI-00014 Helsinki, Finland; 5 Department of Plant Biotechnology, Research Institute of Forests and Rangelands, Agricultural Research, Education and Extension Organization (AREEO), Tehran P.O. Box 14968-13111, Iran

**Keywords:** genetic resources, *Aegilops* spp., SSR markers, biochemical traits, wheat

## Abstract

In the present study, we estimated genetic diversity and population structure in 186 accessions of *Triticum* and *Aegilops* species using 24 simple sequence repeat markers (SSR). Furthermore, an association analysis was performed for antioxidant activities, including guaiacol peroxidase (GPX), ascorbate peroxidase (APX), peroxidase (POX), catalase (CAT), and dry matter (DM) under two control and drought stress conditions. Our findings showed that drought treatment significantly decreased DM, whereas activities of all antioxidant enzymes were increased compared to the control conditions. The results of correlation analysis indicated that, under drought stress conditions, all biochemical traits had a positive and significant association with each other and with dry matter. In the molecular section, the results of the analysis of molecular variance (AMOVA) indicated that the molecular variation within species is more than within them. The dendrogram obtained by cluster analysis showed that grouping the investigated accessions was in accordance with their genomic constitutions. The results of association analysis revealed 8 and 9 significant marker–trait associations (MTA) under control and drought stress conditions, respectively. Among identified MTAs, two associations were simultaneously found in both growing conditions. Moreover, several SSR markers were associated with multiple traits across both conditions. In conclusion, our results could provide worthwhile information regarding marker-assisted selection for the activity of antioxidant enzymes in future breeding programs.

## 1. Introduction

Water availability is one of the most important parameters in determining global agricultural crop productivity [1]. Among abiotic stress, drought is known as one of the most important effects-reducing crop productivity worldwide. It has been reported that inaccessibility to water is responsible for more than 50% of the variance in final plants’ performance. Hence it is the most common source of performance losses throughout the world, and developing more drought-tolerant varieties is one of the key strategies for food security [2]. Plants will be faced with a water deficit at least of some duration in their life cycle; hence, they use several defense mechanisms to survive and adapt to water scarcity conditions [3]. When drought stress is prolonged, it will result in the over-generation of reactive oxygen species (ROS), which in turn cause oxidative stress [4]. ROSs are the result of the partial decrease of atmospheric oxygen (O_2__∙_). These types of oxygen basically have four forms, including singlet oxygen (^1^O_2_), superoxide radical (O_2_^−^), hydrogen peroxide (H_2_O_2_), and hydroxide radical (HO^∙^) [5]. The over-accumulation of these species due to any type of environmental stressors can result in several damages at the cellular and molecular levels, which in turn leads to cell and plant death [6].

Plants have several defense mechanisms that enable them to detoxify over-accumulated ROS. One of these mechanisms is the scavenging system. This system consists of two types of enzymatic and non-enzymatic groups of antioxidants. The first group includes the detoxification product malondialdehyde (MDA), monodehydroascorbate reductase (MDHAR), catalase (CAT), peroxidase (POD), superoxide dismutase (SOD), ascorbate peroxidase (APX), and glutathione reductase (GR). The second group consists of glutathione (GSH), carotenoids, ascorbate acid (AsA), and tocopherols [6]. Of these, enzymatic antioxidants have the key roles in increasing tolerance to various abiotic stresses, and in several types of studies, they are considered biomarkers for the identification of tolerant samples at both the seedling and whole-plant stages [7,8,9,10,11,12,13,14].

Progress in molecular tools and biotechnological approaches have led to the development of the markers associated with any type of plant properties. One of the advanced molecular techniques is association analysis. Over the decades, association analysis has kept its novelty in plant breeding. By discovering relationships between phenotyping and genotyping values within large quantities of networks of data, we can glean insights into many aspects of plant growth and development. In general, this approach has been successfully applied in plant, animal, and human research [15]. In plant breeding, association analysis has been employed for detecting genomic regions for various agronomic traits, biochemical properties, root-system-architecture features, and physiological traits, as well as tolerance to environmental stresses [15,16,17,18,19,20,21,22,23]. As has been proven, the existence of a high level of genetic diversity among the studied samples is one of the basic prerequisites in association analysis. In this regard, the use of local germplasm and/or wild relatives can provide acceptable results.

The wild relatives of crop plants are potentially served as important gene resources for improving world agricultural production and maintaining sustainable agro-ecosystems [13]. The introgression of novel genes from germplasm has been identified as one of the worthwhile strategies for increasing crop tolerance to various abiotic stresses and also improving its productivity [24]. As such, under stressful conditions, breeders must decrease the impact of environmental stresses on final performance through improving the genetic background [25]. Among crop plants, wheat (*Triticum aestivum* L.) has a crucial role in supplying food security and calories consumed by people worldwide. Furthermore, wheat has a huge gene pool, and most studies were focused on its germplasm’s potential [26]. The genera *Aegilops* and *Triticum* are two important members in wheat germplasm, so the species belonging to these genera have significant roles in wheat domestication. Based on numerous studies, any of these species are an ideal source of genes and even alleles related to biotic stress tolerance predominantly for cold, drought, heat, and salinity tolerance [14,22,25,27,28,29,30,31]. A piece of complete information regarding the potential of the wild relatives of wheat was recently reviewed by Pour-Aboughadareh et al. [25]. As a result, all *Aegilops* and *Triticum* species could provide an interesting gene pool for wheat breeders.

Numerous physiological and biochemical studies have been performed on drought tolerance in wheat; however, information regarding the antioxidant capability of this germplasm and also the genomic regions associated with these biochemical features is limited. Hence, the main aim of the present research was to reveal the association between some antioxidant enzymes such as GPX, CAT, APX, and POX with several microsatellite markers (SSR) in a set of wheat accessions under drought stress conditions.

## 2. Materials and Methods

### 2.1. Plant Materials and Experimental Layout

A set of 186 accessions belonging to four species, including *Ae. tauschii* Coss (48 samples), *Ae. cylindrica* Host (47 samples), *T. aestivum* L. (47 samples), and *Ae.s crassa* Boiss (44 samples), were studied for phenotypic and genotypic diversity in some biochemical properties. All samples were provided by the Ilam University Genebank (IUGB) and the National Plant Genebank of Iran (NPGBI) (Supplementary Table S1).

A factorial pot experiment with three replications was carried out in a research glasshouse at the Department of Agronomy and Plant Breeding, Tehran University, Karaj, Iran, in 2018–2019. After breaking seed dormancy, all samples were sown in plastic pots (40 cm height and 20 cm diameter), filled with equal parts soil, humus, and sand. After complete germination, all seedlings were grown under optimal conditions of temperature (25/15 ± 2 °C during the day and night) and photoperiod (16/8 h light/dark). After the appearance of three true leaves, drought stress was initiated based on the field capacity (FC) of each pot. The FC was determined according to the method suggested by Souza et al. [32]. Half of the pots were kept under full field capacity (FC = 95 ± 5%) as the control conditions, and the other half were subjected to drought stress (FC = 30 ± 5%) as the drought stress conditions, for 20 days. After 30 days, the shoot biomass, along with the activities of several antioxidant enzymes, were determined.

### 2.2. Phenotyping Assay

Thirty days after sowing and applying drought stress, leaves of tested samples were subjected to the extraction of crude enzymes based on a method proposed by Pagariya et al. [33]. For this, 100 mg of fresh leaves was homogenized in 1 mL extraction buffer. The extraction buffer contained 0.1 mM EDTA, phosphate buffer (pH 7.4), 0.1% x-Triton, and 1% polyvinyl pyrrolidone (PVP). The extracts were filtered and immediately centrifuged at 15,000× *g* for 20 min at 4 °C. Antioxidant enzyme activities, such as peroxidase (POD), catalase (CAT), guaiacol peroxidase (GPX), and ascorbate peroxidase (APX), were determined based on the separated supernatants as described by Manoranjan and Dinabandhu [34], Hadwan [35], Chance and Maehly [36], and Nakano and Asada [37], respectively. Finally, the shoots of each sample were harvested and oven-dried at 70 °C for 72 h. After that, the dry tissues were weighed and recorded as dry matter (DM).

### 2.3. Genotyping Assay

The total DNA for all investigated samples was isolated based on the CTAB protocol [38]. The quality of isolated DNAs was confirmed using 0.8% agarose gel electrophoresis. To achieve molecular data, a set of microsatellite primers was selected based on the D genome of bread wheat, which was previously developed by Roder et al. [39] (Table 1). All PCR reactions were run out in a 20 μL reaction mixture comprising 10 μL master mix 2XPCR (ready-to-use PCR master mix 2X; Ampliqon), 6 μL double-distilled water, 2 μL of template DNA, and 2 μL of each primer. Amplification reactions were carried out as follows: an initial denaturation step at 95 °C for 5 min, followed by 35 cycles of denaturation at 95 °C for 45 s, a primer-annealing temperature that varied between 51.3 and 69.3 °C for 45 s, and primer elongation at 72 °C for 60 s; the final extension at 72 °C was held for 5 min. The amplified products were visualized on a 2% agarose gel stained with safe view II using a UV-based imaging system.

### 2.4. Data Analysis

A general linear model (GLM) was used to assess the main [drought stress treatments (D) and accessions (A)] and interaction effects (D × A) using R software [40]. The percentage changes in antioxidant profiles and dry biomass were estimated as used by Pour-Aboughadareh et al. [9]. To identify the most tolerant individuals from each species, the stress tolerance index (STI) was calculated using the iPASTIC toolkit [41]. To group the measured traits and to study the relationships among biochemical features with dry biomass, principal component analysis (PCA) was performed using the ‘factoextra’, ‘ggdendro’, and ‘ggplot2’ packages of R software [40].

In the molecular section, all banding patterns obtained from PCR products were used to make the binary matrices. To test the efficiency of used primers, several informativeness parameters such as average heterozygosity (*He*), polymorphism information content (PIC), and gene diversity (*H*) were calculated. An analysis of molecular variance (AMOVA) was done with the aim of the dissection of genetic variation between and within species using the GenAlEx package ver. 6.5 [42]. To compare the level of genetic diversity in the investigated species, some genetic indices, including the number of observed alleles (*Na*), the effective number of alleles (*Ne*), Nei’s gene diversity (*H*), Shannon’s information index (*I*), and the percentage of polymorphic loci (*PPL*), were calculated using GenAlEx package [42]. The phylogenetic tree was drawn based on Jaccard’s genetic similarities coefficients using MEGA ver. 5.1 software [43]. Furthermore, principal coordinate analysis (PCoA) was computed using GenAlEx package [42]. A Bayesian clustering algorithm was used to analyze population structure using STRUCTURE 2.3.4 software [44]. Using a web-based STRUCTURE HARVESTER v2.3.4 [45], the optimum number of subpopulations (Δ*K*) was obtained. A marker–trait association analysis was performed using SSR markers and measured biochemical traits through a mixed linear model (MLM) using TASSEL ver2.1 software [46].

## 3. Results

### 3.1. Phenotypic Variation

**Figure 1:**
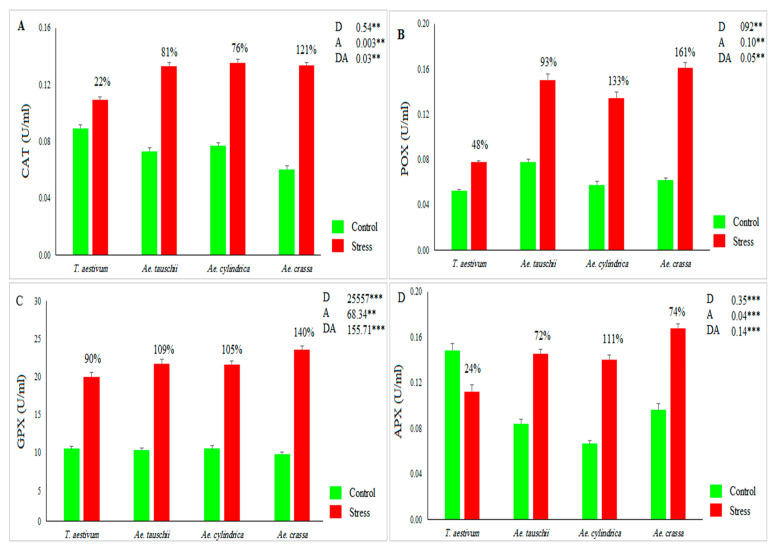
Mean values for (**A**) CAT activity, (**B**) POX activity, (**C**) GPX activity, and (**D**) APX activity across 186 investigated accessions under the control and drought stress conditions. D, A, and DA indicate drought treatment, accessions, and their interaction effects, respectively. ** and *** indicate significance at *p* < 0.01 and *p* < 0.001, respectively.

The results of ANOVA indicated significant variations in growth conditions [control and drought stress environments] and investigated samples for all measured biochemical traits and dry matter (DM). Also, the ‘drought × accession’ interaction effect was significant for all traits except DM. The mean values for CAT activity across all accessions increased under drought stress conditions by 79% compared with the control conditions (Figure 1A). CAT ranged from 0.01 to 0.15 U mL^−1^ in the control conditions and varied between 0.03 and 0.25 U mL^−1^ in stress conditions. Under drought stress conditions, *Ae. cylindrica* showed the highest activity of CAT compared with other species (Figure 1A). Among the tested species, *Ae. crassa* and *Ae. cylindrica* showed the maximum values for this antioxidant enzyme under drought stress conditions (Figure 1A). POX activity ranged from 0.01 to 0.14 with an average of 0.06 U mL^−1^ in the control condition and from 0.05 to 0.32 with an average of 0.13 U mL^−1^ in the stress conditions. Indeed, drought stress increased the POX activity by 109% compared with control conditions. Among species, *Ae. crassa* showed the maximum value for POX under drought stress conditions (Figure 1B).

**Figure 2:**
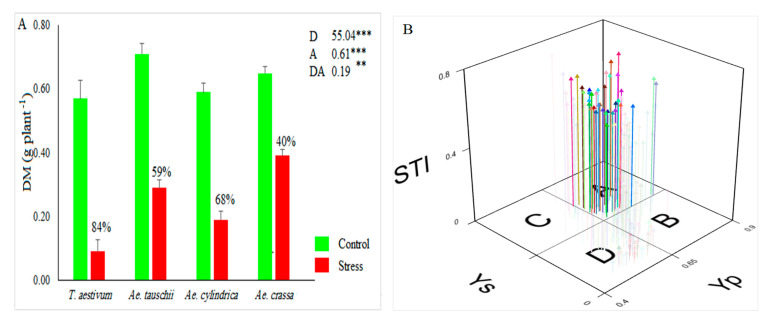
(**A**) Mean value for dry matter (DM) and (**B**) 3D-plot rendered based on dry matters under the control and drought stress conditions (Yp and Ys). ns, **, and *** indicate not significant, significant at *p* < 0.01, and significant at *p* < 0.001, respectively. D, A, and DA indicate drought treatment, accessions, and their interaction effects, respectively.

Like other antioxidant enzymes, the activity of GXP increased from drought stress by 108%. Under control conditions, GXP varied between 4.31 and 31.95, with a mean of 10.35 U mL^−1^, while under stress conditions, it ranged from 9.04 to 36.40, with an average of 21.59 U mL^−1^. Furthermore, the highest activity of GPX was observed for *Ae. crassa* species under drought stress conditions (Figure 1C). The APX activity was significantly affected by drought stress. This enzyme ranged from 0.03 to 0.26, with an average of 0.09 U mL^−1^ under the control conditions, whereas under the drought stress conditions, it varied between 0.03 and 0.29, with an average of 0.14. As a result, drought stress increased the activity of this antioxidant enzyme across all samples by 44.38% when compared with the corresponding values under control conditions. From a species viewpoint, *Ae. cylindrica* showed the highest APX activity under the stress conditions (Figure 1D). As a result, drought stress decreases the DM parameter. Under stress conditions, DM decreased by 62% compared to control conditions (range: 0.48–0.85, with a mean of 0.63 gr in the control, and 0.05–0.51, with a mean of 0.24 g in the stress conditions). *Ae. tauschii* and *Ae. crassa* showed the most DM under control and drought stress conditions, respectively (Figure 2A). The STI index was calculated in order to select the most drought-tolerant accessions. This index varied between 0.07 and 0.75, with an average of 0.39. Based on the 3D plot rendered by STI and dry biomass under control and drought stress conditions (Figure 2B), 41 samples were recognized as Fernandez’s group A. Of these, 22 and 18 samples belonged to *Ae. crassa* and *Ae. tauschii* species, respectively. Furthermore, only one sample from *Ae. cylindrica* was placed in group A.

**Figure 3:**
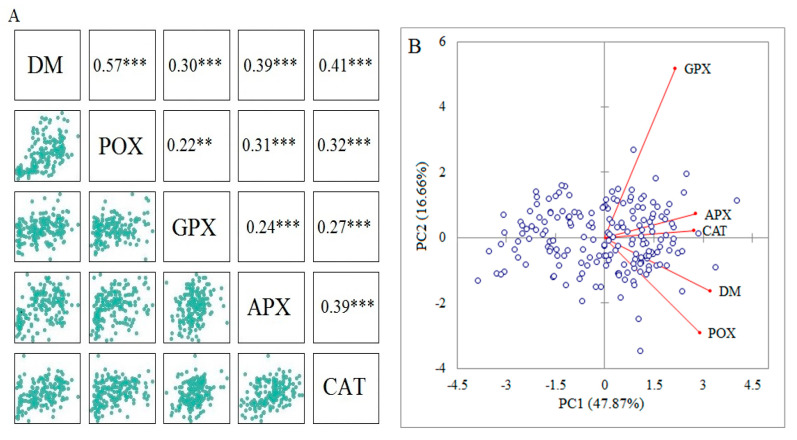
(**A**) The Pearson’s correlation coefficients between the measured biochemical traits and dry matter (DM). (**B**) The PCA-based biplot rendered by the first two components (PC1 and PC2) to show the distribution of investigated accessions based on the measured traits. ** and *** indicate significance at *p* < 0.01, and *p* < 0.001, respectively.

Pearson’s correlation coefficients showed that DM was significantly correlated with all antioxidant activities under drought stress conditions. Hence, these biochemical traits can be used as biomarkers to identify drought-tolerant accessions (Figure 3A). A principal component analysis (PCA) was used to assay the existence of biochemical variation among the instigated wild wheat accessions under drought stress conditions. Accordingly, the first two principal components (PCs) accounted for 64.52% (PC1: 47.86% and PC2: 16.66) of the total biochemical variation. According to the PCA-based biplot, all accessions were widely separated from each other, suggesting a high level of variability among the investigated germplasms (Figure 3B).

### 3.2. Molecular Variation

In this section, 48 polymorphic fragments were generated using the 24 polymorphism SSR primers across all *Triticum* and *Aegilops* accessions.

**Figure 4:**
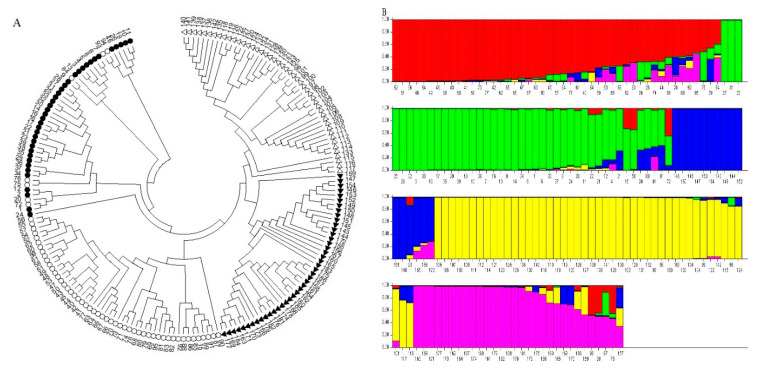
**(A)** The Fan-dendrogram obtained using SSR markers shows the phylogenetic relationships among the investigated wheat accessions. Empty circles, filled circles, empty triangles, and filled triangles indicate *Ae. tauschii*, *T. aestivum*, *Ae. cylindrica*, and *Ae. crassa*, respectively. (**B**) STRUCTURE analysis of the investigated accessions. Bars represent the membership coefficients of samples based on allele frequencies for subpopulations.

The PIC values varied between 0.15 (*Xgwm-272*) and 0.38 (*Xgwm-157*, *Xgwm-469*, and *Xgwm-325*), with an average of 0.32 (Table 1). The average heterozygosity (He) was 0.69, and it ranged from 0.18 (*Xgwm-272*) to 1 (*Xgwm-157*, *Xgwm-469*, and *Xgwm-325*). The gene diversity (H) ranged from 0.16 to 0.50, with an average of 0.41. Among the utilized primers, *Xgwm-16*, *Xgwm-296*, *Xgwm-301*, *Xgwm-325*, *Xgwm-469*, *Xgwm-157*, and *Xgwm-484* showed the highest gene diversity (Table 1). The results of AMOVA revealed that the percentage of variance was higher within species than between them (64% vs. 36%). In terms of genetic variation parameters, *Ae. tauschii* showed the maximum values of Na, Ne, I, He, and PPL compared with other species. To investigate phylogenetic genetic relationships among all investigated accessions, cluster analysis was computed (Table 2). Based on the rendered dendrogram from this analysis, all samples were grouped into four main clear clusters. The two first clusters separately consisted of all *Ae. cylindrica* and *Ae. crassa* accessions. All *Ae. tauschii* accessions (except seven samples) created the third cluster. The fourth cluster included all *T. aestivum* accessions along with the remaining samples of *Ae. tauschii* (Figure 4A).

The SSR data matrix was used for estimating structure analysis. The results of structure analysis showed that the optimum ∆K was 5 (Figure 4B), and the Q matrix was extracted for marker–trait association analysis. The association analysis between biochemical traits and SSR data was computed based on the MLM method. Under control and drought stress conditions, eight and nine significant associations were found, respectively. The coefficient of determination (R^2^) ranged from 2.30 to 13.93% under control conditions, while it varied between 5.86 and 13.70% under stress conditions (Table 3). Under control conditions, the highest *R^2^* values were observed for markers *Xgwm-455*, *Xgwm-484*, and *Xgwm-296*, while markers *Xgwm-455*, *Xgwm-515*, and *Xgwm-608* showed the highest *R^2^* values under drought stress conditions (Table 3). Under control conditions, some SSR markers were significantly associated with DM and enzymatic activities as follows: *Xgwm-212* and *Xgwm-484* with APX; *Xgwm-272*, *Xgwm-296*, and *Xgwm-232* with CAT; *Xgwm-349*, *Xgwm-455*, and *Xgwm-515* with DM. Under drought stress conditions, markers *Xgwm-271* and *Xgwm-608* were significantly associated with APX. The activity of GPX showed a significant association with *Xgwm-455* and *Xgwm-232* markers. The association between *Xgwm-515* and POX was also significant. DM and STI were simultaneously associated with *Xgwm-455* and *Xgwm-515* markers (Table 3). As a part of the results, the *Xgwm-455* marker was simultaneously associated with GPX, DM, and STI under drought stress conditions. Furthermore, *Xgwm-515* was significantly associated with POX, DM, and STI. Under both control and drought stress conditions, markers *Xgwm-455* and *Xgwm-515* showed a significant association with DM (Table 3).

## 4. Discussion

*Aegilops* and *Triticum* species are the most important relatives of bread wheat. Knowledge of genetic diversity in these natural resources can provide an opportunity for future wheat-breeding programs [47]. Despite various studies that have exhibited the excellent breeding potential of these species, their genetic potential is still mainly underutilized [48]. Hence, estimating the population structure in wheat germplasm is one of the main steps of the utilization of breeding potential.

Drought or water-deficit stress is one of the main consequences arising from changes in climate. Thus, screening wheat germplasm in different growth and development stages is an important task for improving its drought tolerance in future breeding programs [49]. Therefore, we assessed the responses of a core collection of bread wheat genotypes and wild relatives possessing the D genome to drought stress at the early stage of growth in terms of dry matter and four antioxidant enzymes. Our results showed a high level of physiological and biochemical variation among the investigated accessions (Figure 1 and Figure 2). This result was in accordance with the previous studies on the response of the wild relatives of wheat to drought stress [11,13,18,50].

As part of the phenotypic assay, highly positive and significant associations were observed among dry matter and activities of CAT, APX, GPX, and POX enzymes under drought stress conditions (Figure 3A). Indeed, this finding confirms the value of the worthiness of the data in the marker–trait association analysis [15]. Multivariate methods provide powerful classifiers to capture phenotypic diversity and can identify separate groups related to functional plant adaptation and regional origin [51]. In this work, principal component analysis (PCA) showed that the biochemical traits and dry matter captured 64.52% of the total variation, suggesting that the antioxidant profiles were efficient in showing the genetic variation among the investigated wheat accessions (Figure 3B). The biplot-based PCA simultaneously indicated the interrelationships among between biochemical profile and dry matter (DM), as well as the distribution of samples based on the two first PCs. The first PC showed a positive and significant association with DM and other biochemical traits; hence, it is named the effective component under drought stress. As shown by the angles among traits’ vectors, all antioxidant enzymes were positively correlated with DM. Formerly, these associations were confirmed by the Pearson’s coefficients (Figure 3B). Likewise, several reports indicated that PCA is an efficient multivariate method to display the level of phenotypic variation and intercept associations between measured traits [9,15,52,53,54,55].

To identify the most drought-tolerant accessions, we used a 3D plot rendered using STI index and dry matter values under both control and drought stress conditions. Among drought-tolerance indices, STI has a good capability of identifying the most tolerant genotypes, and this fact has been confirmed in numerous studies [56,57,58,59]. Accordingly, out of 186 accessions, 41 samples were identified as the most tolerant accessions. All recognized samples (except one accession) belonged to *Ae. crassa* and *Ae. cylindrica* species (Figure 2B).

Furthermore, a high level of molecular variation was also revealed among the investigated wheat accessions through SSR markers. The used primers generated two polymorphic bands across all investigated accessions (Table 1). Based on three marker-informativeness parameters (*PIC*, *He*, and *H*), we found that the used primers had a good efficiency for assessing the genetic diversity and population structure analyses. In other words, this finding reveals an acceptable competence of the used primers in marker–trait association analysis in the investigated germplasm. According to the result of ANOVA, a high degree of molecular diversity was found within species (Table 2). Previously, several researchers estimated the genetic diversity in wheat germplasm through various molecular marker techniques. For instance, Moradkhani et al. [60] using microsatellite markers reported a vast genetic diversity in some *Aegilops* populations. Pour-Aboughadareh et al. [61] using the ISSR technique revealed the high genetic diversity among *Triticum boeoticum* populations collected from different regions of Iran. Furthermore, Naghavi et al. [62], Pour-Aboughadareh et al. [63], Arabbeigi et al. [28], and Etminan et al. [64] reported a high level of genetic diversity among Iranian wild relatives of wheat using various markers such as ISSR, SSR, SCoT, and CBDP markers. As shown in Table 2, the genetic diversity in the *Ae. tauschii* population was greater than what was found among other species. This result suggests a good breeding potential of this species for exploring new genes and even alleles with the aim of utilization in breeding programs. In this regard, numerous studies mentioned the fact that *Ae. tauschii* can serve as an ideal breeding resource especially for improving tolerance to abiotic stresses. Ahmadi et al. [54] examined a set of the wild relatives of wheat and reported that *Ae. tauschii* responded well to drought stress due to its expanded root system. This species has also shown good potential to cope with salt stress when it was investigated under severe salinity conditions at the seedling stage [10].

After detecting a high level of biochemical and molecular variability in the studied germplasm, we used a MLM model-based marker–trait association analysis (MTA) to incorporate phenotypic and genotypic data [65]. The results of association analysis for the biochemical profile and other parameters indicated that the MLM model was effective in detecting significant MTAs. This finding is in agreement with previous reports that showed a high efficiency of the MLM method to perfectly present the significant association [15,16,66,67,68]. In this study, a total of eight and nine significant SSR markers associated with DM and activities of antioxidant enzymes in the control and drought stress conditions, respectively (Table 3). The explained coefficient of determinations (*R*^2^) for the identified MTAs were high (varied between 2.30 and 13.93% in the control and between 5.86 and 13.70% in the drought stress conditions), suggesting many genes contribute to the main part of the measured quantitative traits [15].

Under control conditions, significant MTAs were as follows; *Xgwm-212* and *Xgwm-484* with APX, *Xgwm-272*, *Xgwm-296*, and *Xgwm-232* with CAT, and *Xgwm-349*, *Xgwm-455*, and *Xgwm-515* with DM. Under drought stress conditions, markers *Xgwm-271* and *Xgwm-608* were significantly associated with APX. Two markers *Xgwm-455* and *Xgwm-232* showed a significant association with GPX. The association between *Xgwm-515* and POX was also significant. The DM and STI were simultaneously associated with *Xgwm-455* and *Xgwm-515* markers. Furthermore, the *Xgwm-455* marker was simultaneously associated with GPX, DM, and STI under drought stress conditions (Table 3); this result is more desirable in breeding programs [16]. Among the significant associations, *Xgwm-455* and *Xgwm-515* markers revealed a significant association with DM under both growing conditions. In other words, this finding reveals that the growing conditions are not effective in these MTAs. Indeed, this result confirms that these genomic regions are associated with different growth and development features across two growing conditions. Hence, these markers are important to wheat breeding for biochemical adaptation in drought-prone environments [69].

## 5. Conclusions

Antioxidant enzymes play a key role in the defense of plants and help them to cope with the oxidative stress induced by various types of environmental stresses. The identification of molecular markers associated with these biochemical parameters can accelerate the screening of tolerant genetic materials in breeding programs. The results of this study illustrated that wild relatives of wheat have a high level of biochemical and molecular variability. Furthermore, the SSR marker system was a powerful technique for identifying significant marker–trait associations in this germplasm. Our results indicated that the interaction between growth conditions and accessions is not influenced by some MTAs. Therefore, the results obtained from the present study contribute to the knowledge of genetics and breeding for key biochemical activities in wheat.

## Figures and Tables

**Table 1:** Estimated informativeness parameters for the utilized SSR markers in the present study.

Primer	Chromosome Position		Sequence (5′ to 3′)	*Na*	*He*	*H*	*PIC*
Xgwm-16	5D	Forward	GCTTGGACTAGCTAGAGTATCATAC	2	0.93	0.50	0.37
		Reverse	CAATCTTCAATTCTGTCGCACGG				
Xgwm-44	7D	Forward	GTTGAGCTTTTCAGTTCGGC	2	0.73	0.46	0.36
		Reverse	ACTGGCATCCACTGAGCTG				
Xgwm-111	7D	Forward	TCTGTAGGCTCTCTCCGACTG	2	0.28	0.24	0.21
		Reverse	ACCTGATCAGATCCCACTCG				
Xgwm-121	5D & 7D	Forward	TCCTCTACAAACAAACACAC	2	0.88	0.49	0.37
		Reverse	CTCGCAACTAGAGGTGTATG				
Xgwm-271	5D	Forward	CAAGATCGTGGAGCCAGC	2	0.59	0.41	0.33
		Reverse	AGCTGCTAGCTTTTGGGACA				
Xgwm-272	5D	Forward	TGCTCTTTGGCGAATATATGG	2	0.18	0.16	0.15
		Reverse	GTTCAAAACAAATTAAAAGGCCC				
Xgwm-292	5D	Forward	TCACCGTGGTCACCGAC	2	0.42	0.33	0.28
		Reverse	CCACCGAGCCGATAATGTAC				
Xgwm-296	2D	Forward	AATTCAACCTACCAATCTCTG	2	0.92	0.50	0.37
		Reverse	GCCTAATAAACTGAAAACGAG				
Xgwm-301	2D	Forward	GAGGAGTAAGACACATGCCC	2	0.99	0.50	0.37
		Reverse	GTGGCTGGAGATTCAGGTTC				
Xgwm-325	6D	Forward	TTTCTTCTGTCGTTCTCTTCCC	2	1	0.50	0.38
		Reverse	TTTTTACGCGTCAACGACG				
Xgwm-349	2D	Forward	GGCTTCCAGAAAACAACAGG	2	0.97	0.50	0.37
		Reverse	ATCGGTGCGTACCATCCTAC				
Xgwm-382	2D	Forward	GTCAGATAACGCCGTCCAAT	2	0.84	0.49	0.37
		Reverse	CTACGTGCACCACCATTTTG				
Xgwm-455	2D	Forward	ATTCGGTTCGCTAGCTACCA	2	0.70	0.45	0.35
		Reverse	ACGGAGAGCAACCTGCC				
Xgwm-469	6D	Forward	CAACTCAGTGCTCACACAACG	2	1	0.50	0.38
		Reverse	CGATAACCACTCATCCACACC				
Xgwm-515	2D	Forward	AACACAATGGCAAATGCAGA	2	0.36	0.29	0.25
		Reverse	CCTTCCTAGTAAGTGTGCCTCA				
Xgwm-565	5D	Forward	GCGTCAGATATGCCTACCTAGG	2	0.71	0.46	0.35
		Reverse	AGTGAGTTAGCCCTGAGCCA				
Xgwm-583	5D	Forward	TTCACACCCAACCAATAGCA	2	0.73	0.46	0.36
		Reverse	TCTAGGCAGACACATGCCTG				
Xgwm-608	2D & 5D	Forward	ACATTGTGTGTGCGGCC	2	0.77	0.47	0.36
		Reverse	GATCCCTCTCCGCTAGAAGC				
Xgwm-624	4D	Forward	TTGATATTAAATCTCTCTATGTG	2	0.65	0.44	0.34
		Reverse	AATTTTATTTGAGCTATGCG				
Xgwm-157	2D	Forward	GTCGTCGCGGTAAGCTTG	2	1	0.50	0.38
		Reverse	GAGTGAACACACGAGGCTTG				
Xgwm-212	5D	Forward	AAGCAACATTTGCTGCAATG	2	0.57	0.41	0.33
		Reverse	TGCAGTTAACTTGTTGAAAGGA				
Xgwm-232	1D	Forward	ATCTCAACGGCAAGCCG	2	0.24	0.21	0.19
		Reverse	CTGATGCAAGCAATCCACC				
Xgwm-311	2D	Forward	TCACGTGGAAGACGCTCC	2	0.77	0.47	0.36
		Reverse	CTACGTGCACCACCATTTTG				
Xgwm-484	2D	Forward	ACATCGCTCTTCACAAACCC	2	0.95	0.50	0.37
		Reverse	AGTTCCGGTCATGGCTAGG				
			Mean	1.96	0.69	0.41	0.32

*Na*, *He*, *H*, and *PIC* indicate the number of amplified alleles, average heterozygosity, gene diversity, and polymorphism information content, respectively.

**Table 2:** Estimated genetic variation parameters using SSR markers in four investigated wheat germplasm.

Species	*Na*	*Ne*	*H*	*I*	*PPL*	Variation between Species	Variation within Species
*T. aestivum*	1.65 ± 0.09	1.47 ± 0.06	0.27 ± 0.03	0.40 ± 0.04	73.47	64%	36%
*Ae. tauschii*	1.87 ± 0.05	1.66 ± 0.05	0.37 ± 0.02	0.53 ± 0.03	89.80		
*Ae. cylindrica*	1.24 ± 0.09	1.16 ± 0.04	0.10 ± 0.02	0.16 ± 0.03	38.78		
*Ae. crassa*	1.41 ± 0.10	1.31 ± 0.05	0.18 ± 0.03	0.28 ± 0.04	53.06		
Mean	1.45 ± 0.05	1.40 ± 0.03	0.23 ± 0.01	0.34 ± 0.02	63.78		

*Na*, observed number of alleles; *Ne*, Effective number of alleles; *H*, Nei’s genetic diversity; *I*, Shannon’s information index; *PPL*, percentage of polymorphic loci.

**Table 3:** The list of significant marker–trait associations under the control and drought stress conditions.

Control Conditions				Drought Stress Conditions			
Trait	Marker	*p*	R^2^%	Trait	Marker	*p*	R^2^%
APX	*Xgwm-212*	0.049	8.15	APX	*Xgwm-271*	0.021	6.18
	*Xgwm-484*	0.009	13.79		*Xgwm-608*	0.006	11.51
CAT	*Xgwm-272*	0.046	7.18	GPX	*Xgwm-455*	0.019	8.10
	*Xgwm-296*	0.002	13.03		*Xgwm-232*	0.014	7.38
	*Xgwm-232*	0.010	6.51	POX	*Xgwm-515*	0.023	8.45
DM	*Xgwm-349*	0.044	6.79	DM	*Xgwm-455*	0.005	13.70
	*Xgwm-455*	0.0001	13.94		*Xgwm-515*	0.008	13.18
	*Xgwm-515*	0.0001	8.89	STI	*Xgwm-455*	0.013	6.84
					*Xgwm-515*	0.037	9.71

## Data Availability

Not applicable.

## Supplementary Materials

The following supporting information can be downloaded at: https://www.mdpi.com/article/10.3390/genes13081491/s1, Table S1: Passport of the genetic materials used in this study.
