# Supplementary material for: Association Analysis for Some Biochemical Traits in Wild Relatives of Wheat under Drought Stress Conditions
Source: Genes (Basel). 2022 Aug 21;13(8):1491. doi: 10.3390/genes13081491 (PMC9408274; doi:10.3390/genes13081491)
Supplement: Supplementary file 1 [file genes-13-01491-s001.zip › genes-1837979-supplementary.pdf]

Supplementary Table S1. Passport of the genetic materials used in this study.

No.	Species	Code	No.	Species	Code	No.	Species	Code	No.	Species	Code
1	TA	†7499-2	48	AT	NPGBI-01-0836	95	AT	NPGBI-01-1559	142	AC	IUGB-00388S
2	TA	IUGB-00133	49	AT	IUGB-00020	96	AC	IUGB-00373	143	ACR	NPGBI-976
3	TA	IUGB-00134	50	AT	IUGB-00107	97	AC	IUGB-00189	144	ACR	NPGBI-365
4	TA	IUGB-00264	51	AT	IUGB-00164	98	AC	IUGB-00236	145	ACR	NPGBI-310
5	TA	IUGB-00447	52	AT	IUGB-00193	99	AC	IUGB-00267	146	ACR	NPGBI-300
6	TA	IUGB-00453	53	AT	IUGB-00196	100	AC	IUGB-00188	147	ACR	NPGBI-309
7	TA	IUGB-00460	54	AT	IUGB-00198	101	AC	IUGB-00359	148	ACR	NPGBI-2066
8	TA	IUGB-00480	55	AT	IUGB-00039	102	AC	IUGB-00403	149	ACR	NPGBI-1589
9	TA	IUGB-00482	56	AT	IUGB-00223	103	AC	IUGB-00210	150	ACR	NPGBI-792
10	TA	IUGB-00485	57	AT	IUGB-00224	104	AC	IUGB-00185	151	ACR	NPGBI-947
11	TA	IUGB-00516	58	AT	IUGB-00245	105	AC	IUGB-00156	152	ACR	NPGBI-1485
12	TA	IUGB-00911	59	AT	IUGB-00247	106	AC	IUGB-01746	153	ACR	NPGBI-1508
13	TA	IUGB-01569	60	AT	IUGB-00260	107	AC	IUGB-00221	154	ACR	NPGBI-384
14	TA	IUGB-01635	61	AT	IUGB-00261	108	AC	IUGB-00200	155	ACR	NPGBI-2112
15	TA	IUGB-01671	62	AT	IUGB-00143	109	AC	IUGB-00150	156	ACR	NPGBI-1384
16	TA	IUGB-01695	63	AT	IUGB-00325	110	AC	IUGB-00168	157	ACR	NPGBI-720
17	TA	IUGB-01696	64	AT	IUGB-00365	111	AC	IUGB-00034	158	ACR	NPGBI-2063
18	TA	IUGB-00615	65	AT	IUGB-00366	112	AC	IUGB-00078	159	ACR	NPGBI-911
19	TA	IUGB-00597	66	AT	IUGB-00369	113	AC	IUGB-00090	160	ACR	NPGBI-1699
20	TA	IUGB-00604	67	AT	IUGB-00402	114	AC	IUGB-00406	161	ACR	NPGBI-587
21	TA	IUGB-00603	68	AT	IUGB-00151	115	AC	IUGB-00258	162	ACR	NPGBI-794
22	TA	IUGB-00576	69	AT	IUGB-00291	116	AC	IUGB-00248	163	ACR	NPGBI-944
23	TA	IUGB-00618	70	AT	IUGB-00382	117	AC	IUGB-00388	164	ACR	NPGBI-2117
24	TA	IUGB-01845	71	AT	IUGB-00238	118	AC	IUGB-01592	165	ACR	NPGBI-1742
25	TA	IUGB-00518	72	AT	IUGB-00249	119	AC	IUGB-00271	166	ACR	NPGBI-598
26	TA	IUGB-00593	73	AT	IUGB-00367	120	AC	IUGB-00202	167	ACR	NPGBI-1638
27	TA	IUGB-00570	74	AT	IUGB-00273	121	AC	IUGB-00201	168	ACR	NPGBI-744
28	TA	IUGB-00575	75	AT	IUGB-00274	122	AC	IUGB-00406	169	ACR	NPGBI-1473
29	TA	IUGB-01846	76	AT	IUGB-00276	123	AC	IUGB-00229	170	ACR	NPGBI-1522
30	TA	IUGBI-00577	77	AT	IUGB-00279	124	AC	IUGB-00090	171	ACR	NPGBI-675
31	TA	IUGBI-00589	78	AT	IUGB-00289	125	AC	IUGB-00270	172	ACR	NPGBI-730
32	TA	IUGB-00573	79	AT	IUGB-00374	126	AC	IUGB-00059	173	ACR	NPGBI-689
33	TA	IUGB-00600	80	AT	IUGB-00383	127	AC	IUGB-00132	174	ACR	NPGBI-50067
34	TA	IUGB-00578	81	AT	IUGB-00386	128	AC	IUGB-00095	175	ACR	NPGBI-50119
35	TA	IUGB-00602	82	AT	IUGB-00396	129	AC	IUGB-00062	176	ACR	NPGBI-50174
36	TA	IUGB-00586	83	AT	IUGB-00400	130	AC	IUGB-01359	177	ACR	NPGBI-50040
37	TA	IUGB-00598	84	AT	IUGB-00401	131	AC	IUGB-01238	178	ACR	IUGB-00379
38	TA	IUGB-00515	85	AT	IUGB-00404	132	AC	IUGB-00239	179	ACR	IUGB-01564
39	TA	IUGB-01847	86	AT	IUGB-00405	133	AC	IUGB-00078	180	ACR	IUGB-00881
40	TA	IUGB-00534	87	AT	NPGBI-01-1970	134	AC	IUGB-00065	181	ACR	IUGB-00817
41	TA	IUGB-00613	88	AT	NPGBI-01-2120	135	AC	IUGB-00391	182	ACR	IUGB-00170
42	TA	IUGB-00590	89	AT	IUGB-00297	136	AC	IUGB-00241	183	ACR	IUGB-00408
43	TA	IUGB-00606	90	AT	IUGB-01746	137	AC	IUGB-00153	184	ACR	IUGB-00319
44	TA	IUGB-00599	91	AT	NPGBI-50006	138	AC	IUGB-00390	185	ACR	IUGB-00280
45	TA	IUGB-01840	92	AT	NPGBI-50084	139	AC	IUGB-00399	186	ACR	IUGB-00284
46	TA	IUGB-00532	93	AT	NPGBI-01-0312	140	AC	IUGB-00201S			
47	TA	IUGB-00580	94	AT	NPGBI-01-0804	141	AC	IUGB-01592			

TA, AT, AC and ACR indicate *T. aestivum*, *Ae. tauschii*, *Ae. cylindrica* and *Ae. crassa* species respectively.

IUGB: Ilam University Genebank

NPGBI: The National Plant Gene Bank of Iran

†Tehran University Genebank
